# Material Extrusion of Wool Waste/Polycaprolactone with Improved Tensile Strength and Biodegradation

**DOI:** 10.3390/polym15163439

**Published:** 2023-08-17

**Authors:** Abu Naser Md Ahsanul Haque, Maryam Naebe

**Affiliations:** Institute for Frontier Materials, Deakin University, Geelong, VIC 3216, Australia; a.haque@deakin.edu.au

**Keywords:** bio-composite, fused deposition modelling, 3D printing, natural wool, filament extrusion, sustainability

## Abstract

Additive manufacturing (AM) through material extrusion (MEX) is becoming increasingly popular worldwide due to its simple, sustainable and safe technique of material preparation, with minimal waste generation. This user-friendly technique is currently extensively used in diverse industries and household applications. Recently, there has been increasing attention on polycaprolactone (PCL)-based composites in MEX due to their improved biodegradability. These composites can be printed at a lower temperature, making them more energy efficient compared to commercial filaments such as acrylonitrile butadiene styrene (ABS) and polylactic acid (PLA). Although wool is the leading protein fibre in the world and can be more compatible with PCL due to its inherent hydrophobicity, the suitability of MEX using a wool/PCL combination has not been reported previously. In the current study, waste wool/PCL composite parts were printed using the MEX technique, and rheology, thermal and tensile properties, and morphology were analysed. The impact of wool loading (10% and 20%) was investigated in relation to different filling patterns (concentric, rectilinear and gyroid). Furthermore, the impact of fibre fineness on the final material produced through MEX was investigated for the first time using two types of wool fibres with diameters of 16 µm and 24 µm. The yield strength and modulus of PCL increased with the inclusion of 10% wool, although the elongation was reduced. The crystallinity of the composites was found to be reduced with wool inclusion, though the melting point of PCL remained mostly unchanged with 10% wool inclusion, indicating better compatibility. Good miscibility and uniform structure were observed with the inclusion of 10% wool, as evidenced by rheology and morphology analysis. The impact of fibre fineness was mostly minor, though wool/PCL composites showed improved thermal stability with finer diameter of wool fibres. The printed specimens exhibited an increasing rate of biodegradation in marine water, which was correlated to the amount of wool present. Overall, the results demonstrate the practical applicability of the wool/PCL composition in MEX for the preparation of varied objects, such as containers, toys and other household and industrial items. Using wool/PCL combinations as regular plastics would provide a significant environmental advantage over the non-degradable polymers that are currently used for these purposes.

## 1. Introduction

Material extrusion (MEX), also known as fused deposition modelling (FDM), has gained significant attention in recent years as a unique and sustainable method of materials fabrication. It is a simple concept of additive manufacturing (AM) that is free from hazardous chemicals [1], and allows for the safe fabrication of complex and elegant structures in a tabletop environment. This method is also recognised for its negligible waste generation, low production cost [2], and ability to deliver materials with a higher stress/weight ratio [3]. Over the years, MEX has been adopted in numerous sectors, including composites [4], scaffolds [5], energy harvesting [6], pharmaceuticals [7], and home applications [8]. Moreover, MEX is playing a significant role in the recycling and recovery of different thermoplastic composites [9,10,11]. 

In the MEX method, a thermoplastic filament is guided through a heated nozzle that moves in horizontal and vertical directions as per a pre-planned design. The molten filament extrudes on a print bed in a controlled manner through the nozzle to produce the final material [12]. 

Recently, with the increase in global awareness of cleaner manufacturing methods and sustainable materials, polycaprolactone (PCL) has been gaining significant attention in MEX due to its confirmed biocompatibility and lower processing temperature compared to common commercial MEX filaments, such as acrylonitrile butadiene styrene (ABS) and polylactic acid (PLA). PCL has proven biodegradability in the natural environment, contrasting the non-degradability of ABS and a temperature requisite of above 50 °C for the successful degradation of PLA [13]. Moreover, ABS and PLA need a higher extrusion temperature (around 200–220 °C) [14], whereas PCL can be printed at a lower temperature (such as 80–150 °C) [15] and can be sourced from renewable resources cost-effectively [16]. Focusing on these advantages, numerous studies have been performed in recent years on MEX of pure PCL [17] as well as PCL composites, such as PCL/PLA [18], PCL/hydroxyapatite [5], PCL/thermoplastic polyurethane [19], PCL/polyglycolic acid [20], PCL/PBAT (poly butyleneadipate-co-terephthalate) [4], PCL/beta-tricalcium phosphate [21], PCL/gum rosin/beeswax [15], PCL/silk [22], PCL/cocoa shell waste [14] and so on.

Although PCL has shown evidence of biodegradation, its degradation rate is slow. The complete degradation of PCL may take 4 years or more, while an apparent difference can be observed at least after a year [23]. To promote the degradation rate of PCL, natural fillers are often used, which can bring significant differences. However, PCL is extremely hydrophobic, which results in poor compatibility with most natural fillers that are hydrophilic [24]. Even though biodegradation can be accelerated, other important properties required for the practical application of PCL composite may have a negative impact. 

An alternative option to ensure faster degradation of PCL using fillers and maintain good compatibility in the three-dimensional (3D) printed materials could be the use of natural hydrophobic fillers, such as protein fibres including wool or silk. Due to the lack of polarity in these fibres, they are not as repulsive as the natural cellulosic fibres towards PCL, and can produce a more compatible structure, eluding the efforts for polymer grafting [25] or compatibilisation [26]. 

Wool is the leading protein fibre obtained from animals widely around the world. As per a recent International Wool Textile Organisation (IWTO) report, worldwide production of raw wool is currently 1.95 million tonnes, resulting in ~1 million tonnes of clean wool [27]. This is significantly higher than other animal protein fibres, such as down (0.53 million tonnes), silk (0.11 million tonnes) and cashmere (0.025 million tonnes) [28]. Apart from the wool waste produced during its cleaning (i.e., the remaining 0.95 million tonnes of the 1.95 million tonnes), wool is collected as waste (12–15% waste) during its processing into yarn and fabric and as consumed textile waste [29]. Moreover, using wool as a filler in PCL can improve the biodegradation rate since wool itself is biodegraded within 6 months in appropriate conditions [30]. 

The physicochemical structure of wool fibres provides it with some unique properties. Though chemically, wool mainly consists of keratin [31], it is also composed of cortical cells, cell-membrane complex and cuticular cells. The inner core of the fibre is shielded by overlying layers of cuticle cells, i.e., epicuticle, endocuticle and exocuticle. The outer layer of this fibre is enriched with lipids that are interconnected to surface proteins and cell-membrane complex [32]. The complex presence of lipids generates hydrophobicity in wool, which is often retained even after chemical processing, such as Soxhlet extraction [33]. Therefore, wool could be a potential candidate as a natural filler into PCL for MEX, as the hydrophobicity of wool would be an advantage during blending and extrusion with hydrophobic PCL, compared to other common natural fillers. Earlier study has confirmed that the wool/PCL combination exhibits good compatibility, as evidenced by the minor change in the position of the melting enthalpy peak in composite with the addition of 10% wool, compared to pure PCL [34]. Typically, a less compatible combination would result in a greater shift of the melting peak to a lower temperature, indicating easier melting of the polymer. Further, the low melting temperature of PCL was proven as an advantage for keeping the chemical structure of wool intact while extruding. Moreover, both wool and PCL are biodegradable, thus as a combination more sustainable and environmentally friendly compared to the common polymers used in 3D printing. 

However, to date, there has been no previous study on the combination of wool and PCL in MEX. Though the properties of the pre-printing material (i.e., wool/PCL filaments) were reported [34], the changes in the characteristics of the final material after 3D printing were not assessed. This study aims to demonstrate the impact of different amounts of wool, wool fibre fineness and filling patterns on the 3D-printed wool/PCL composites. Worthy of note, this is also the first report that investigates the impact of the initial fineness of any fibrous filler in MEX. Printing was performed after the preparation of wool/PCL filaments, and rheology, morphology, and thermal and tensile properties were investigated. Three different patterns, namely concentric, rectilinear and gyroid, were chosen, as the former two were reported to produce better tensile strength in the literature [35], and the latter is preferred by the manufacturer (Prusa Research) and claimed to have a good strength/weight ratio [36]. The results are discussed in terms of their interrelation as well as in relation to the properties of filaments discussed in previous study.

## 2. Materials and Methods

This study adopted a clean production method, minimising the generation of any in-process residue. The materials used and the methods applied for the wool/PCL 3D printing are described in the following subsections. 

### 2.1. Materials

Two undyed wool fabric waste products weaved from 16.1 μm and 23.8 μm were collected from Commonwealth Scientific and Industrial Research Organisation (CSIRO), Geelong Waurn Ponds Campus, Australia. Commercial polycaprolactone (CAPA6800) polymer granules (Mw 80,000) were purchased from Era Polymers, Banksmeadow, Australia. Sodium hydroxide (NaOH) pellet was purchased from Chem-Supply, Gillman, Australia.

### 2.2. Powder Preparation

The particle size of the filler in 3D printing is preferable in micro-level to maintain good mixing and avoid clogging in the 3D printer nozzle. Therefore, wool fabrics were first cut into a coarse form by a cutting mill (Pulverisette 19, Fritsch, Idar-Oberstein, Germany) using a 1 mm mesh. Two different wool coarse powders collected by cutting were then individually milled for 1.5 h using an S/1 attritor mill (Union Process, Akron, OH, USA) having ceramic balls of 5 mm diameter. The collected slurries were filtered under a vacuum to obtain wet powders. The wet powders were then dried in ambient temperature (~20 °C), and the final powders were obtained by grinding in a ring grinder (Pulverisette 14, Fritsch, Germany). The average particle size (d50), measured using a laser diffraction technique (Mastersizer 3000, Malvern, PA, USA), for the powders from 16.1 μm was 22.2 μm and from 23.8 μm wool was 46.3 μm. The detailed volume-based particle size distributions of these two powders are available in a previous study [34].

### 2.3. Preparation of Filaments and Printing

Wool powders were separately blended with PCL pellets in a twin-screw extruder (Wayne, NJ, USA) to formulate wool/PCL pellets. There were two hoppers in the machine, the main hopper and the side hopper, used for separate input of PCL and wool. The feeding speed of wool powders was adjusted to 0.22 kg/h and 0.5 kg/h to obtain 10/90 and 20/80 wool/PCL combinations, respectively. A further high amount of wool was not considered due to breakage and poor results reported in the previous study [34]. The temperatures from Zone 1 to 6 were increasingly set from 82 to 93 °C and 96 °C for the die zone following previous wool/PCL extrusion work [34]. The extruded wool/PCL was passed through a water bath for cooling, and wool/PCL pellets were collected through a pelletiser. 

The prepared filaments were then extruded through a desktop extruder (Wellzoom, Shenzhen Mistar Technology Co., Ltd, Shenzhen, China) to obtain the 3D printable filament. The temperature was maintained at 95 °C for preheating and 90 °C for extrusion stages [34]. The filaments were prepared at 2000 mm/min speed through a 1.75 mm nozzle, resulting in the final diameter of 1.40 ± 0.05 mm. A control filament was also prepared using the same parameter as the pure PCL pellets.

**Figure 1:**
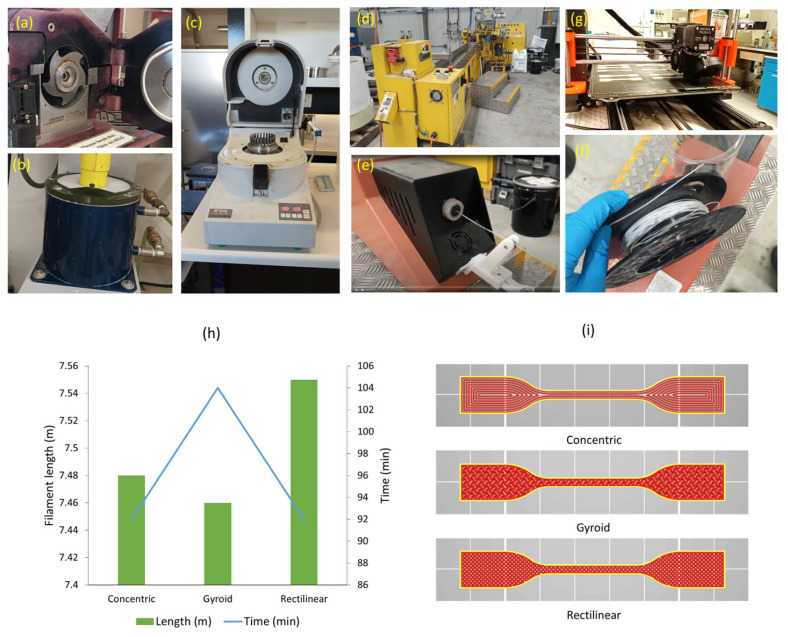
(**a**) Cutting machine, (**b**) attritor tank, (**c**) ring grinder, (**d**) twin-screw extruder used for pellet making, (**e**) filament extruder, (**f**) produced filament, (**g**) 3D printing with filament, (**h**) differences in filament length and printing time across different patterns when printing 10 samples at once, and (**i**) patterns used for infill.

The 3D printing of dog-bone shapes was performed as per ISO 527-2 [37] 5A by an i3 MK3 3D printer (Prusa Research, Prague, Czech Republic) using a 0.4 mm nozzle. The printing temperature was tested and adjusted to 130 °C to obtain the optimised printability from the composite filaments. The printing bed temperature was set to 25 °C. A 50% infill was used to print specimens of concentric, rectilinear and gyroid patterns, while top and bottom fill patterns for all of the samples were monotonic. A 45° fill angle was used for the printing, and the speed of infill was 80 mm/min, though the first layer speed was set to 20 mm/min. The nomenclature of the printed specimens is shown in Table 1. Figure 1 shows the equipment used from powder preparation to 3D printing along with the designs used for printing and time, and filament consumption differed across filling patterns. 

### 2.4. Characterisations

The 3D-printed samples were characterised by their rheology, thermal properties, tensile properties, morphology, and biodegradation properties. The techniques used for characterising the produced samples are detailed in the following subsections.

#### 2.4.1. Rheology

The rheology of pure PCL and the wool/PCL printed combination was tested at 130 °C, which was the optimised 3D printing temperature. Samples were tested in a discovery HR3 rheometer (TA instruments, New Castle, DE, USA) using a 40 mm diameter parallel-plate geometry. The geometry gap was set to 1 mm, and the test was performed from 0.1 to 100 rad/s at 1% strain.

#### 2.4.2. Thermal Properties

Derivative thermogravimetry (DTG) of the samples was measured using a TGA Q50 (TA Instruments, USA) in N_2_ atmosphere. The test was conducted from 30 to 600 °C, maintaining a 20 °C/min heating rate. The graph was plotted using the derivative weight change (%) of the samples per degree of temperature rise on the y-axis and the change in the temperature on the x-axis.

Differential scanning calorimetry (DSC) of the samples was performed using a DSC Q200 (TA Instruments, USA) instrument in N2 atmosphere. The test was conducted from 30 to 600 °C using a 20 °C/min heating rate. The differences in enthalpy at the endothermic stages were measured in TA Universal analysis software. The PCL crystallinity index (CI) was calculated using Equation (1) [34]:

CI = (ΔH_x_)/(ΔH_100_) × 100 (1)

where ΔH_x_ and ΔH_100_ are the enthalpy change of a sample and the theoretical enthalpy change of 100% crystalline PCL (139 J/g) [38], respectively.

#### 2.4.3. Tensile Properties

The yield strength, yield strain, breaking stress, breaking strain, and modulus of elasticity of the ISO 527-2 5A-sized printed dog-bone samples were assessed using a universal tensile testing system (Instron 5967, Norwood, MA, USA) fitting with a 1 kN load cell and 50 mm/min elongation rate. The specific yield stress, specific breaking stress and specific modulus of the samples were determined by dividing the yield stress, breaking stress, and modulus values by the density of the specimen.

Three specimens of each sample were tested, and average and standard deviation were reported. The significance of the difference between the datasets was determined by a two-tailed *t*-test. The confidence level was considered as 95%, and thus, *p* ≤ 0.05 was denoted as a significant difference, and *p* > 0.05 was denoted as no significant difference.

#### 2.4.4. Morphology

The morphology of the cross-section of the samples was examined as normal and after the tensile experiment (fractured cross-section). Samples were coated with platinum in an EM ACE600 sputter coater (Leica, Macquarie Park, Australia), and scanning electron microscope (SEM) images were taken by a Supra 55 VP (Zeiss, Oberkochen, Germany) instrument.

#### 2.4.5. Biodegradation

**Figure 2:**
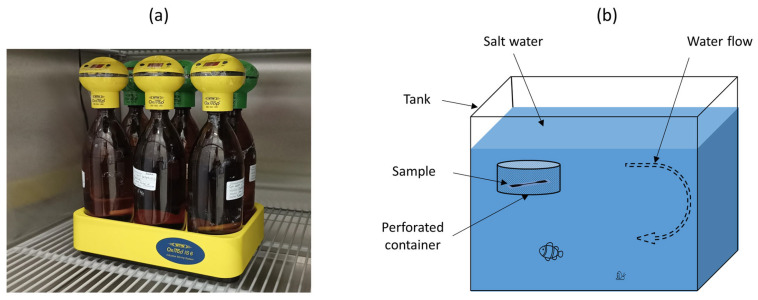
(**a**) BOD test progressing inside a refrigerated incubator, and (**b**) a schematic of replication of marine environment in a saltwater fish tank.

The biodegradation of the composites and pure PCL samples was measured in two methods. In the first method, it was indirectly measured by monitoring the biochemical oxygen demand (BOD) in marine water following the BS EN 1899-2:1998 protocol [39]. The marine water collected from Eastern Beach, Geelong, Australia (latitude 38.15° S, longitude 144.37° E, altitude 21 m) was considered in the experiment. The test was conducted using an OxiTop BOD system (WTW, Xylem Analytics, Letchworth, UK.), containing 6 bottles. In each bottle, 432 mL of marine water was used. Around 50 ± 1 mg of printed sample was inserted in five bottles and one bottle was left without any sample (only water). The generated carbon dioxide (CO_2_) was absorbed by sodium hydroxide from the BOD closed system. The equipment was placed inside a refrigerated incubator (Thermoline Scientific, Wetherill Park, Australia), and the temperature was maintained constant at 20 °C (Figure 2a), and experiment ran for 5 days as per the WTW instruction manual for OxiTop instrument. The actual BOD was calculated by subtracting the value of the blank sample (marine water without any sample). The test was repeated three times, and average data were considered for each sample.

In the second method, the marine environment was replicated in a domestic saltwater fish tank as an established ecosystem. The volume of water was 140 L, constantly circulated at a 2000 L/h flow rate (Figure 2b). The temperature, pH and specific gravity of the water were maintained at 25 °C, 8.1–8.3, and 1.025, respectively. The ammonia and nitrite contents were 0 mg/L, and the nitrate content in water was <10 mg/L (measured using API marine test kit, Mars, Chalfont, PA, USA). The samples were placed inside the tank in a perforated container so that the water could pass through the samples. After 5 months, the samples were taken out, and the loss of weight was measured in relation to their initial weight.

## 3. Results

The results obtained from the tests were found interlinked with each other and were affected by the wool amount in the composites, the initial fineness of wool, as well as the 3D printing patterns. The findings are detailed in the following subsections. 

### 3.1. Rheology

**Figure 3:**
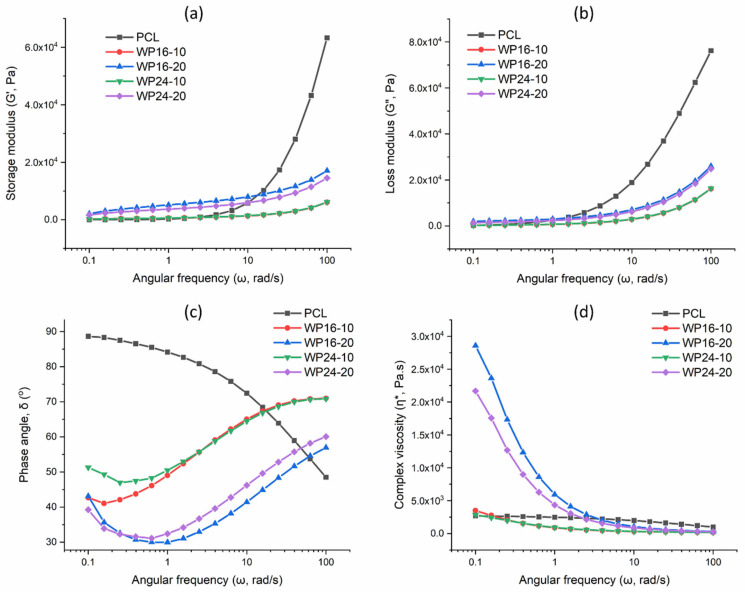
Rheological properties of polycaprolactone (PCL), wool/PCL (WP) composites prepared from 16 and 24 µm wool at 130 °C: (**a**) storage modulus, (**b**) loss modulus, (**c**) phase angle, and (**d**) complex viscosity.

Figure 3a shows the storage modulus (G′) of PCL and wool/PCL composites at 130 °C. Commonly, a higher storage modulus is associated with higher stiffness of a material [40]. The G′ value of the samples was seen increasing with the increase in angular frequencies. For example, G′ of PCL ranged around 6.3 to 61.4 Pa at lower frequencies (i.e., 0.1–0.4 rad/s), though at higher frequencies it was around 10,210–63,280 Pa (i.e., 15–100 rad/s). This behaviour is due to the reduced amount of time to respond to the stress at higher frequencies, and the material tends to show more stiff behaviour than viscous [34]. The changes in the viscoelastic behaviour of PCL and wool/PCL composites were more apparent at lower frequencies.

At frequencies around 0.1–0.4 rad/s, G′ of the composites prepared with 10% wool ranged around 179–442 Pa, though a significantly higher amount of G′ was found (around 1678–4220 Pa) for the samples loaded with 20% wool. This showed the increased amount of stiffness produced in the PCL polymer chain by the influence of the increased wool amount. The curves for the composites were more flat compared to the pure PCL, demonstrating more frequency-independent characteristics as the wool/PCL interactions probably restricted the chain mobility [40]. For MEX, this behaviour is also linked to the improved ability of the extruded material to hold its shape as printed [15]. 

Figure 3b illustrates the loss modulus (G″) of the samples. The loss modulus is mainly linked to the dissipation of energy from a material when stress is applied. At lower frequencies, there was a clear difference observed between samples prepared with 10% and 20% wool. Even though pure PCL showed a radical increase in the loss modulus with increasing frequencies, at a lower frequency (0.1 rad/s) the value (269 Pa) was mostly identical to those of samples prepared from 10% wool (223–236 Pa) and very different from the samples prepared from 20% wool (1372–1955 Pa). This indicated that up to 10% wool was enough to preserve the energy, maintaining appropriate miscibility between wool and PCL [34], though when 20% wool was included, friction among the particles increased, and more vacant spots were produced in the interfaces; this helped the energy dissipation [40]. At the higher frequencies, more energy dissipated from all of the samples, probably more related to the PCL chain behaviour (as seen from the pure PCL curve), rather than the wool particles. In cases of both storage and loss moduli, the percentage of wool particles showed a major impact on the viscoelastic characteristics of the composites, though the fineness of the initial wool showed a minor influence. 

Figure 3c shows the phase angle (δ) value of the samples. The tangent of phase angle is known as the loss factor, which is the slope of the G′-G″ curve. When δ > 45° (tan δ > 1), the material behaviour is more inclined to be viscous, and if δ < 45° (tan δ < 1), the material behaviour is more inclined to be solid-like. A clear influence of the wool reinforcement was seen on tan δ, compared with pure PCL, which showed a complete viscous characteristic (δ = 48.5–88.6°) at the tested frequencies, under 130 °C. This was probably because of the stiffness of the wool itself compared to PCL, which significantly affected the δ values. A lower δ value, of mostly around 45° or below, was persistent up to ~1 rad/s for samples with 10% wool, and up to ~10 rad/s for samples with 20% wool. This aligns with the improved and consistent storage modulus seen earlier in the composite samples, higher for samples with the higher percentage of wool. The change in δ from <45° to >45° in most of the composite samples was mainly related to the transition from the viscoelastic plateau to the transition region of a typical viscoelastic curve [41].

Figure 3d represents the complex viscosity (η*) of the samples, again showing a major influence from the amount of wool loading, i.e., higher η* from greater wool loading. The overall decrease in η* with rising frequencies indicated the shear thinning behaviour in all of the samples [22]. The drop in η* for pure PCL was from 2691 to 990 Pa.s, while it was from around 2861–3489 Pa.s to around 172–174 Pa.s for 10% wool-loaded samples and from around 21,684–28,590 Pa.s to around 287–310 Pa.s for 20% wool-included composite samples. The massive drop in the η* in the 20% wool-included samples was probably related to their less consistent structure due to the presence of more fillers. This is discussed further in the morphology section. The higher η* value of these samples compared to other samples at lower frequencies was probably related to the structural interruption in PCL by wool, producing more voids (can be observed from the morphology discussions later), making the overall system less resistant to flow [22]. At lower frequency (0.1 rad/s), the values of η* were mostly similar among PCL and composites with 10% wool, indicating good miscibility. In terms of initial fibre fineness of wool, a finer fibre mostly showed a higher η*, probably associated with a lower particle size of WP16 compared to WP24 in a similar mass of powder, and the distribution of particles was more uniform in PCL, resulting in more resistance to PCL flow. 

Overall, the rheological behaviour of wool/PCL (particularly with 10% wool) in this study (130 °C) was in some cases closer to that of pure PCL. This was not observed in the previous study with wool/PCL, where only the filament property was investigated at 90 °C [34]. The higher temperature used in the current study (adjusted as per the actual printing requirement) reasonably improved the flowability of the wool/PCL system and reduced the difference in rheological behaviour with pure PCL. 

### 3.2. Thermal Properties

**Figure 4:**
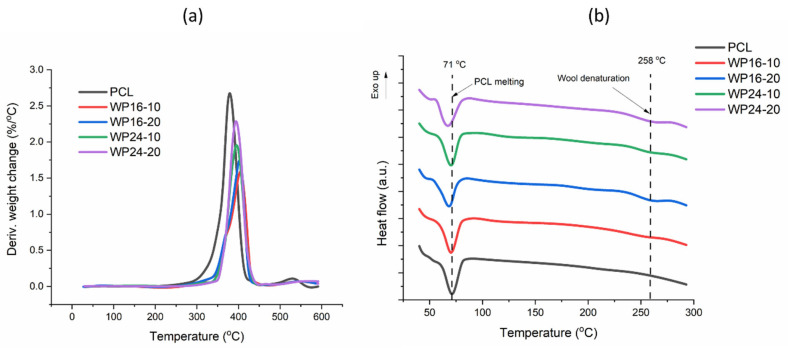
DTG (**a**) and DSC spectra (**b**) of polycaprolactone (PCL) and wool/PCL (WP) composites prepared from 16 and 24 µm wool.

The DTG spectra of pure PCL and the wool/PCL composite samples are shown in Figure 4a. Both PCL and wool/PCL composites showed minor weight loss up to 200 °C, confirming their strong hydrophobic nature. The position of the major DTG peak was shifted to a higher degree when wool was included with PCL; the DTG peak was seen for pure PCL at 379 °C, and then the peak of wool/ PCL composites shifted to 394 °C for coarser and 404 °C for finer wool. The onset of the peak was around 270 °C for PCL, while the onset peak of the composites was around 350 °C. These are clear indications of the improvement in thermal stability of PCL when wool particles have been included.

Between the wools with different fineness, the position of the main peak was higher for the finer diameter of wool (WP16), compared to the coarser diameter of wool (WP24). This indicates that the distribution of finer wool in the PCL matrix was probably more uniform, due to the smaller size of the particles [34]. This can also be verified by the percentages of weight loss of these samples from 270 °C to 450 °C (Table 2), showing a higher weight loss for the WP24 composite samples. The thermal degradation of WP16 samples started earlier than that of WP24 samples, which also showed a shoulder peak before the final peak. This early degradation could be related to cysteine degradation, as this is associated with the cuticle content in the outer layer of wool, and finer wool commonly possesses a higher cuticle proportion than coarser wool [42].

The thermal degradation behaviour of the composites was also related to the wool amount in the composite samples, as higher stability (lower peak height) was seen with 10% wool rather than 20% wool. This indicates that the effectivity of wool in increasing the thermal stability of PCL is better with a lower loading, as the distribution of particles is likely to be more uniform. Though a minor weight loss difference was seen in the overall region (270 to 450 °C), the residue left at 600 °C was higher for the 20% wool-loaded samples, as this residue was more related to wool rather than pure PCL (0.09%).

The DSC spectra of PCL and wool/PCL composites are shown in Figure 4b. The melting peak of pure PCL was seen at 70.9 °C, which was almost unchanged by 10% wool loading (70.3 °C for WP16 and 70.2 °C for WP24). The melting peak shifted towards a lower temperature (~68 °C) when 20% wool was reinforced (Table 1). This indicates that the compatibility between 10% wool and PCL was adequate to retain the melting behaviour of PCL, though a higher amount of wool (20%) produced more imperfections and vacant spots in the interfaces [43]. The intermolecular interactions within the system were obstructed, and PCL started to melt at a lower temperature [44]. The values of melting enthalpy, as well as crystallinity index, showed that the overall crystalline property of PCL was reduced by wool inclusion. The crystallinity index of pure PCL was found in the range of values reported in the literature (nearly 48%) [45]. Crystallinity was decreased around 5.2–5.6% by 10% wool and around 11.4–14.5% by 20% wool. This was similar to findings in previous wool/PCL studies, such as for wool/PCL filaments [34] and wool/PCL nano nets from electrospinning [46]. This is related to the increase in irregular spots in the PCL matrix due to the inclusion of wool, affecting the PCL orientation. 

The denaturation peak of wool due to its α-helix was seen in all of the composites near 258 °C, proving the presence of wool in the structure [29]. Both the wool/PCL composite samples with 20% wool (regardless of the fibre fineness), resulted in a higher value of enthalpy (2.51–2.66 J/g) compared to the samples prepared with 10% wool (0.47–0.49 J/g). This was expected, as more wool was present in those samples, showing a higher denaturation from the wool structure. 

Overall, the thermal degradation and crystalline behaviour of the samples in this study were mostly similar to those observed in the previous study with wool/PCL filaments [34]. This indicates that the thermal property was not altered much after 3D printing, even though the material passed through a slightly higher temperature (130 °C) during the printing operation. 

### 3.3. Tensile Properties

The tensile behaviour of the 3D-printed samples printed in three different patterns (concentric, gyroid and rectilinear) can be divided into three sections. These include the impact on the samples up to the yield point, the impact on the samples up to the breaking point, and the overall impact on their modulus. These are discussed in the following subsections. 

#### 3.3.1. Impact on Yield 

**Figure 5:**
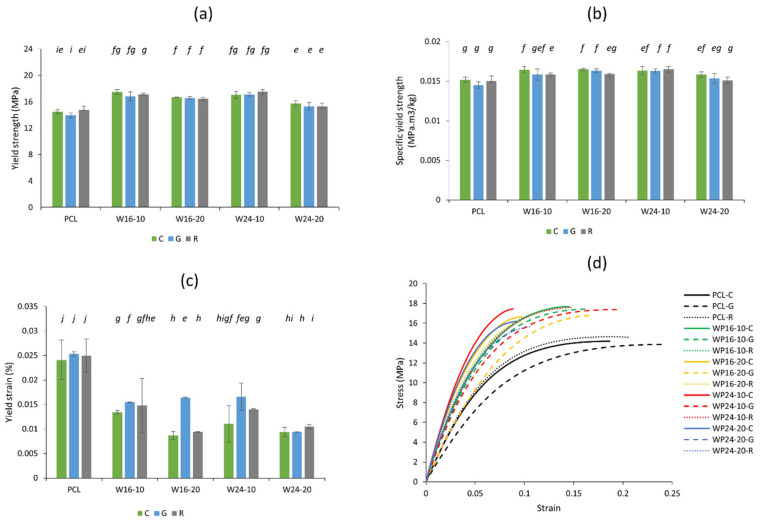
Tensile yield of 3D-printed PCL and wool/PCL composites prepared with 10% and 20% wool with finer (16 µm) and coarser fibre (24 µm), using concentric (C), gyroid (G) and rectilinear (R) infill patterns; (**a**) yield strength, (**b**) specific yield strength, (**c**) yield strain, and (**d**) representative curves of the samples up to the yield point. Different alphabetic letters in italics showing data are significantly different from each other (*p* ≤ 0.05).

The yield point is the first point on a stress–strain curve of a material where strain increases without a rise in stress [47]. This is also known as the elastic limit of the material after which plastic deformation starts and the material cannot come back to its original shape if stress is removed. Figure 5a shows that the yield strength of pure PCL specimens was around 13.9–14.5 MPa, which aligns with commercial PCL (CAPA6800) (yield strength of 14–16 MPa) [48]. The strength was significantly increased (around 15.8–20.6%) when 10% wool was included with PCL, regardless of the fibre fineness [49]. A further increase in wool loading, i.e., 20%, slightly reduced the yield strength, which became more evident with the coarser wool fibre (W24-20). This effect was found to be similar to previously reported tensile properties of wool/PCL filaments of similar loading [34]. The impact of filling patterns was observed as marginal and showed mostly insignificant differences (*p* > 0.05) when loading percentage and fibre fineness were kept constant. The average values were observed to be mostly higher for concentric infill compared to the other two, probably due to its orientation with the tensile loading direction [35].

The 3D-printed parts included in the current study were structures within the specimen, having differences in their internal gaps, influencing density. This can also be seen in Figure 1i, where the filament weights and lengths were measured differently for different infill patterns, as they follow different paths to produce the infill. The strength in relation to the respective density of the material can provide further confirmation of the yield strength results, which are shown in Figure 5b. A general increase in the specific yield strength values by wool loading compared to that of pure PCL, as well as a higher strength for concentric patterns, was evident, validating the results in Figure 4a.

The yield strain values showed sporadic results among the composite samples (Figure 5c), though when wool was loaded, the decrease in elongation was consistent compared to pure PCL. This was because of the decrease in chain deformability of PCL due to the barriers produced in the PCL chain by the addition of wool particles [50]. Though the average yield strain of the concentric-filled specimens was often lower than that of the corresponding samples, differences were often not significant (*p* > 0.05). Figure 5d shows representative stress–strain curves for the samples, the higher elongation property of the pure PCL, but a higher yield stress value for most of the composites.

Overall, the yield strength of the PCL and wool/PCL 3D-printed specimens in this study showed statistically insignificant differences (*p* > 0.05) compared to that of the filaments (15 MPa for PCL filaments and 15.1 to 17.1 MPa for the composites) reported in the previous study [34]. Moreover, the impact of wool loading and fibre fineness observed in this study was mostly consistent with the trends obtained in the case of filaments [34]. 

#### 3.3.2. Impact on Ultimate Break

**Figure 6:**
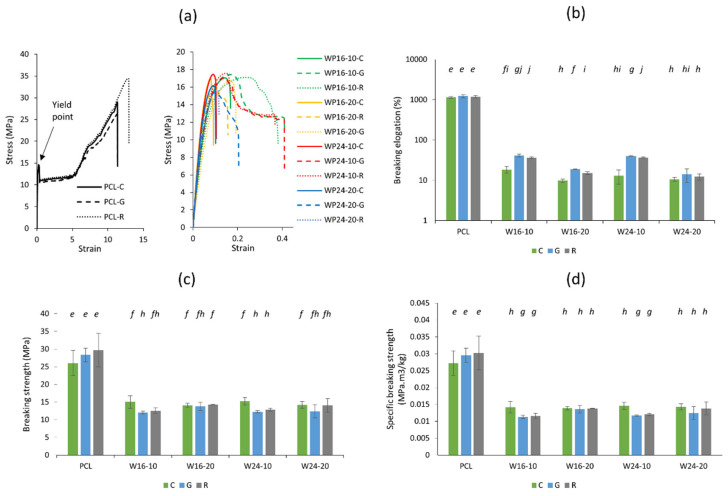
Representative stress–strain curves (**a**) of 3D-printed PCL (**left**) and wool/PCL composites (**right**) prepared with 10% and 20% of wool with finer (16 µm) and coarser fibre (24 µm), using concentric (C), gyroid (G) and rectilinear (R) infill patterns; and (**b**) breaking elongation, (**c**) breaking strength, and (**d**) specific breaking strength of the same samples. Different alphabetic letters in italics showing data are significantly different from each other (*p* ≤ 0.05).

In contrast to the yield point, the impact of wool on the breaking strength and breaking elongation was perceived differently. Figure 6a shows representative curves for the PCL-only samples and the composite samples, indicating a huge alteration in the elastic property of PCL by wool inclusion. After the yield point, PCL showed a high extensibility (1149–1237%) (Figure 6b), similar to that observed in a previous study (>1000%) [51]. The breaking elongation of PCL was observed to be lower than that of a previously reported PCL filament (1780%), probably due to the printed infill that produced void spaces inside the structure. Impacted by the interruption of PCL chain mobility by wool powders, the composite samples showed a drastic reduction in the breaking elongation, to only about 9.9–40% across all samples. Among the composites, composite samples with 10% wool showed higher elongation compared to the samples prepared with 20% wool. This was because of the higher amount of particles present in the 20% wool-loaded samples, creating more disruptions and voids in the PCL chain. The concentric infill showed a lower elongation behaviour, mostly statistically significant (*p* < 0.05) compared to the other patterns in at least three of the four composite samples. A lower elongation in the concentric pattern was found to be similar to that in the previous study with different infill patterns through MEX [52]. 

The breaking strength of pure PCL was 26.1–29.7 MPa (Figure 6c), which was also higher than that of the composite samples (12–15.1 MPa) and explained by the specific breaking strength results (Figure 6d). The breaking strength values of PCL-only samples were higher than their corresponding yield strength values, while the opposite was seen for the wool/PCL composites. Though the breaking strength of the PCL was significantly higher due to the inherent extensibility of the PCL chain, the calculation for the breaking strength may not be meaningful past their yield point, as a massive reduction in the cross-section occurred due to stress after yield (also known as the necking behaviour) [53]. Further, as per the ASTM guide, these values only have a qualitative function after the yield point, as the impact of necking on the entire gauge length before the break is uncertain [47]. This necking behaviour was not seen in any of the composites, while they mostly broke just after the yield point. In cases of both breaking strength and specific breaking strength, the differences among the composites were mostly insignificant with some exceptions, such as a higher strength obtained by concentric infill with 10% wool, which is consistent with the yield strength result for the same sample in this study as well as reported in the literature [35]. 

#### 3.3.3. Impact on Modulus

**Figure 7:**
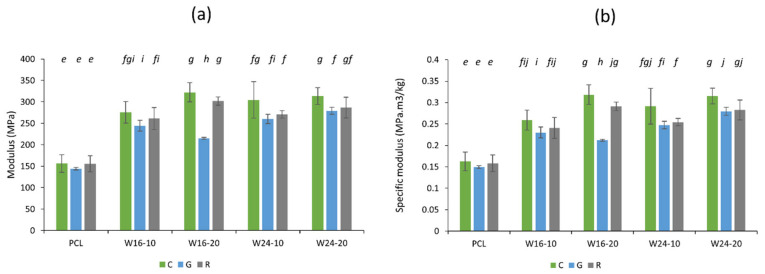
Modulus (**a**) and specific modulus (**b**) of 3D-printed PCL and wool/PCL composites prepared with 10% and 20% wool with finer (16 µm) and coarser fibre (24 µm), using concentric (C), gyroid (G) and rectilinear (R) infill patterns. Different alphabetic letters in italics showing data are significantly different from each other (*p* ≤ 0.05).

The modulus and specific modulus of all samples are shown in Figure 7a,b, respectively. The increase in the modulus and specific modulus by wool inclusion in PCL was evident. This was because of a higher modulus of the wool itself (3.9–5.9 GPa) [54] compared to PCL (i.e., 144–156 MPa in the current study), which increased the rigidity of the composite specimens and is consistent with the literature [55]. The modulus value tended to further increase with the increased filler amount. For example, for the concentric patterns, the modulus increased from 275–304 MPa to 313–322 MPa, in cases of 10% and 20% wool, respectively. The gyroid-infilled specimens showed consistently lower average values for all of the samples, which could be related to the structure of the gyroid infill, which consumed a lower amount of materials and had a reduced amount of mass to resist the force [56]. Except for the general impact of wool on PCL, the differences in the modulus or specific modulus values were often not statistically significant and showed mostly a sporadic result. 

Overall, the tensile strength of PCL composites was comparatively lower than that of the commercial products, such as PLA and ABS (52.5 MPa and 38.1, respectively); however, through the specimen preparation, it was proven that they were stiff enough to retain their designed structure. The composites also showed a lower modulus behaviour compared to commercial PLA and ABS (3250 MPa and 1962 MPa, respectively), which indicates the higher flexibility of the wool/PCL combination, which is also vital for 3D printing [34]. 

### 3.4. Morphology

The morphology of cross-sections of the samples was investigated. This was tested in two phases, after the printing and after the tensile tests (fractured point). The findings are detailed in the following subsections.

#### 3.4.1. Cross-Sections of Printed Specimens

**Figure 8:**
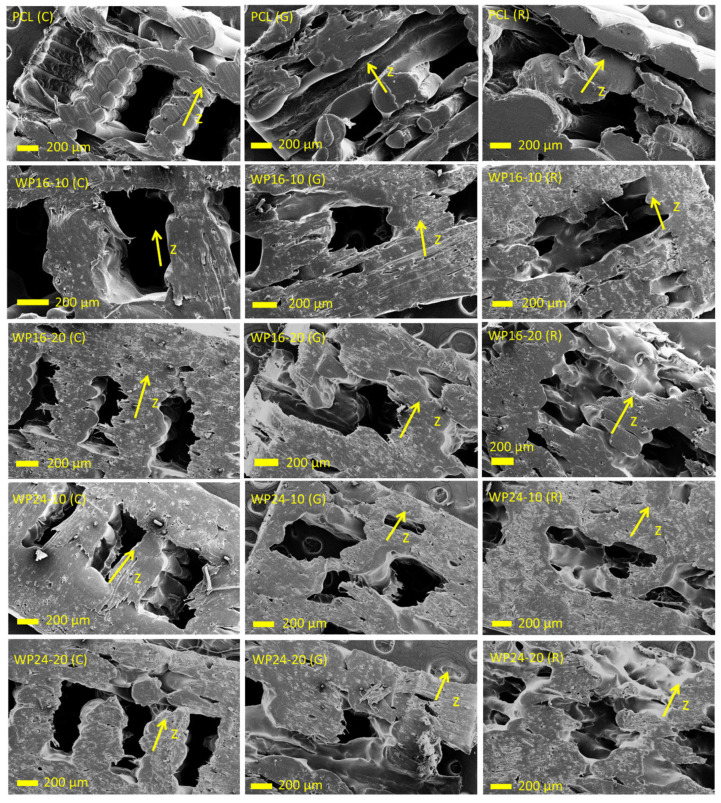
Cross-sections of the 3D-printed specimens, polycaprolactone (PCL), wool/PCL (WP) composites prepared from 16 and 24 µm wool using concentric (C), gyroid (G) and rectilinear (R) infill patterns. Direction of arrows indicating direction of z axis during printing.

The cross-section morphology of the 3D-printed PCL and wool/PCL combinations is shown in Figure 8. Pure PCL showed mostly smooth orientation, and layers were visible in the z direction in all infill patterns. All of the wool/PCL samples showed roughness in their structure due to the reinforcement with wool, though the distribution of wool was perceived to be uniform. This was probably due to good compatibility between wool and PCL, as mentioned during the discussions of rheological and thermal behaviour of these samples. Interestingly, though the presence of the layers was sometimes evident in the composite samples, they were often not as visible as in the pure PCL. This could be due to grasping among the layers, interlocking with each other. 

The patterns of infill were clearer in the PCL samples, though not exactly retained after the printing in the cases of both PCL and the composites. The concentric infill patterns appeared steadier compared to the other two patterns, while PCL composites with 10% wool showed more shape retention ability than those with 20% wool. Even though a higher amount of wool enhanced the rigidity of the composites (a higher modulus), the shape retention ability appeared to be more related to a balanced mixing of wool (i.e., 10%) with PCL, which did not produce many voids inside the matrix [34]. Further, a higher wool loading, as well as coarser wool (i.e., W24-20), showed more inconsistent structures, related to more disturbances in PCL by wool inclusion. 

#### 3.4.2. Cross-Sections of Fractured Specimens

**Figure 9:**
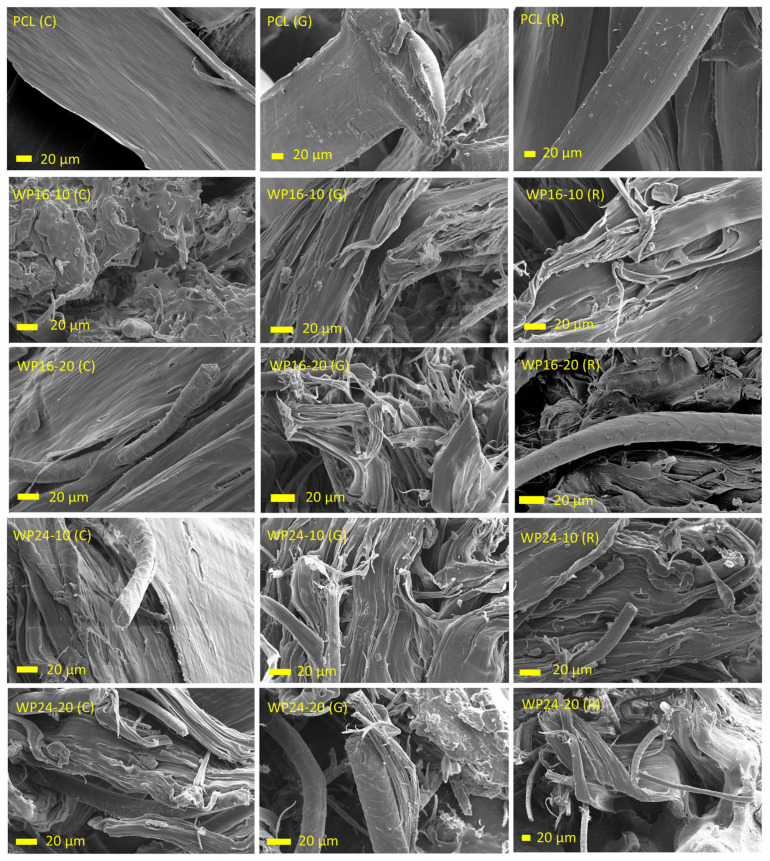
Cross-sections of the tensile tested specimens at the breaking point, polycaprolactone (PCL), wool/PCL (WP) composites prepared from 16 and 24 µm wool using concentric (C), gyroid (G) and rectilinear (R) infill patterns.

The cross-sections at the breaking points of the specimens after the tensile tests are shown in Figure 9. Pure PCL showed a smooth surface and a compact structure of the matrix. As expected, wool/PCL samples showed rougher structure due to the inclusion of fibres that disrupted the matrix orientation and produced voids inside the PCL. This was more observed with 20% wool compared to 10% wool. Two different fineness grades of wool were visible from the scalebar of the samples prepared with 16 µm and 24 µm wool; both preserved their surface scales, indicating the retention of their hydrophobic nature. Other than the fineness, the appearance was mostly identical for these samples, though a greater void area was perceived for the WP24-20 samples, which aligned with a lower yield strength (Figure 4a) observed in the sample, compared to other composite samples. The morphology was not affected much by fibre fineness when a lower loading was used (e.g., 10%), but the differences became more prominent when 20% wool was used instead. This was likely as more fillers produced greater instability in the matrix, and the structure embraced more voids in the wool/PCL interfaces. These trends in the findings were similar to those observed for the morphology of wool/PCL filaments [34], showing unchanged behaviour after printing. Among the infill patterns, the morphology of the fractured gyroid infilled samples showed slightly more void areas. This also aligned with a slightly lower strength value observed in those samples. The possible reason could be a lower density of these samples, which was related to a lower filament consumption (Figure 1h) by the gyroid infill when producing a similarly sized specimen. Overall, comparatively fewer voids in the fractured areas of concentric patterns is consistent with earlier studies with different infill patterns [35]. 

### 3.5. Biodegradation

**Figure 10:**
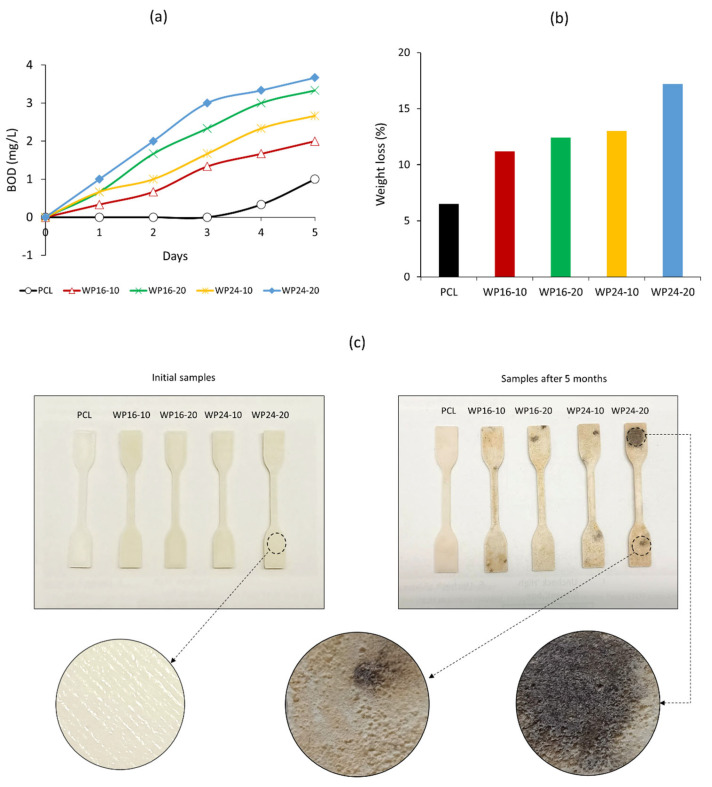
(**a**) BOD results from the biodegradation of polycaprolactone (PCL) and wool/PCL (WP) composites prepared from 16 and 24 µm wool, (**b**) loss of weight of the samples during 5 month biodegradation, and (**c**) biodegraded samples in the marine tank.

In the previous study of wool/PCL filament [34], biodegradation was tested through soil burial. However, to promote widespread use of biodegradable plastics, it is important to study their degradation behaviour in marine environments since significant percentages of plastic waste and microplastics end up in the ocean [57]. The increase in BOD value is an indication of the biodegradation of a sample, as BOD represents the amount of oxygen that microorganisms require to break down a biodegradable material. Figure 10a shows a trend of increasing BOD values related to composite samples compared to pure PCL during the initial 5 days. This indicates that the inclusion of wool with PCL was advantageous for promoting biodegradation. The difference between WP16 and WP24 samples was minor when using the same loading of wool. BOD values were observed to be higher with a higher amount of wool, which further indicated wool’s involvement in fostering the biodegradation property of the composite.

Figure 10b shows the loss of weight of the sample during a 5 month biodegradation period. All of the samples showed a similar trend, as found in BOD testing; a higher weight loss was related to a higher reinforcement with wool. Coarser wool showed a higher rate of degradation, probably related to the presence of a lower amount of cuticles, as reported in the literature [42]. Figure 10c shows the physical changes in the samples during this degradation, with more black spots observed when coarser wool was used in a higher amount (i.e., WP24-20). It was also clear that the printed pattern on the surface was entirely changed during this degradation (higher magnification). The surfaces of the samples became rougher, and random beads were developed, indicating the operation of microorganisms through the sample. Overall biodegradation results confirmed that outside the soil environment, the wool/PCL combination is also biodegradable in the marine environment, which could be an additional advantage of using wool/PCL composites in regular applications. 

## 4. Conclusions 

In this study, 3D printing of waste wool/PCL combinations is proposed as a viable alternative to current commercial polymers. The influence of two different grades of wool fineness (16 μm and 24 μm) was observed on different wool/PCL combinations (10/90 and 20/80), while the impact of filling patterns was also analysed. The overall findings could be summarised as follows-

- The crystallinity index of PCL decreased from 48.1% to around 45.4–45.6% and 41.1–42.6% with 10% and 20% wool, respectively.
- The melting peak position of PCL and composites remained similar for 10% wool inclusion, indicating good compatibility. A finer diameter of initial wool was found to be responsible for producing better thermal stability in the composites.
- The complex viscosity of the samples with 10/90 wool/PCL was identical to that of pure PCL at a lower angular frequency. The cross-sectional morphology of the samples showed that the disruption in the PCL matrix was more related to the loading amount, rather than fibre fineness.
- Among different infill patterns (concentric, rectilinear, gyroid), overall, the concentric infill resulted in higher average strength and lower average elongation, while gyroid infill showed higher average elongation and lower average strength, though often these differences were not significant (*p* > 0.05). Higher yield strength and breaking strength were consistent across the samples reinforced with 10% wool, compared to pure PCL.
- The printed materials showed an increasing trend of BOD and loss of weight in marine water due to biodegradation, corresponding to the amount of wool in the samples.

The results indicate the possible practical use of wool/PCL combinations for MEX of numerous materials, such as household and industrial tools, boxes, containers and more. Future research could be focused on the performance of the developed materials in real-life applications and further improve the sustainability of the process through optimisation, starting from powder preparation to final printing. 

## Figures and Tables

**Table 1:** Nomenclature of the 3D-printed specimens based on wool fineness and wool loading.

Wool Fineness (μm)	Wool (%)	PCL (%)	Name
-	0	100	PCL
16	10	90	WP16-10
16	20	80	WP16-20
24	10	90	WP24-10
24	20	80	WP24-20

**Table 2:** DTG and DSC data for the printed PCL and wool/PCL samples at different temperature stages.

		PCL	WP16-10	WP16-20	WP24-10	WP24-20
Degradation (270–450 °C)	Peak (°C)	379	404	404	394	394
	Weight loss (%)	93.3	86.8	87.1	91.5	91.9
Residue (%)	600 °C	0.09	1.7	1.9	1.5	1.8
Evaporation	Peak (°C)	70.9	70.3	68.4	70.2	67.5
	Enthalpy (J/g)	66.9	63.4	59.2	63.1	57.1
	Crystallinity index (%)	48.1	45.6	42.6	45.4	41.1
Denaturation	Peak (°C)	-	258.8	258.3	258.5	258.1
	Enthalpy (J/g)	-	0.47	2.51	0.49	2.66

## Data Availability

The data presented in this study are available on request from the corresponding author.
